# Luminescent turn-on detection of Hg(II) via the quenching of an iridium(III) complex by Hg(II)-mediated silver nanoparticles

**DOI:** 10.1038/s41598-017-03952-x

**Published:** 2017-06-15

**Authors:** Jinshui Liu, Kasipandi Vellaisamy, Guanjun Yang, Chung-Hang Leung, Dik-Lung Ma

**Affiliations:** 1 Department of Chemistry, Hong Kong Baptist University, Kowloon Tong, Hong Kong, China; 2 State Key Laboratory of Quality Research in Chinese Medicine, Institute of Chinese Medical Sciences, University of Macau, Macao, China

## Abstract

A novel luminescent turn-on detection method for Hg(II) was developed. The method was based on the silver nanoparticle (AgNP)-mediated quenching of Ir(III) complex **1**. The addition of Hg(II) ions causes the luminescence of complex **1** to be recovered due to the oxidation of AgNPs by Hg(II) ions to form Ag(I) and Ag/Hg amalgam. The luminescence intensity of **1** increased in accord with an increased Hg(II) concentration ranging from 0 nM to 180 nM, with the detection limit of 5 nM. This approach offers an innovative method for the quantification of Hg(II).

## Introduction

Heavy metal contamination is a serious hazard to human health and the environment^1–3^. According to the U.S. Environmental Protection Agency (EPA) and the International Agency for Research on Cancer, heavy metals are classified as probable human carcinogens^4^. Among several heavy metal ions, sensing of mercuric (Hg(II)) ions has attracted growing attention due to their acute toxicity^5^. Hg(II) can accumulate in vital organs through the food chain, posing a severe threat to the health of humans and animals^6^. Therefore, monitoring the levels of Hg(II) in aquatic ecosystems is of great significance. Currently, various techniques including colorimetry^7, 8^, surface plasmon resonance (SPR)^9^, electrochemistry^10^ and surface-enhanced Raman scattering (SERS)^11^ have been extensively utilized for sensing Hg(II)^12–15^. Viewed as an alternative approach to these methods, luminescence assays possess promising advantages, such as rapid response, high sensitivity, and simple manipulation^16–18^. Pioneer studies have reported many luminescent probes, including organic molecules^19^, quantum dots (QDs)^20^, and metal nanoclusters^21, 22^ for the detection of Hg(II). Nevertheless, most of these methods show drawbacks, including poor selectivity and sensitivity, complicated synthetic procedures, low stability in aqueous media and a turn-off signal output^20–22^, leading to strong desire of developing alternative approaches. In such a case, luminescence turn-on sensors are generally more desirable than turn-off sensors, as they are less susceptible to false positive signals^23–26^.

**Figure 1:**
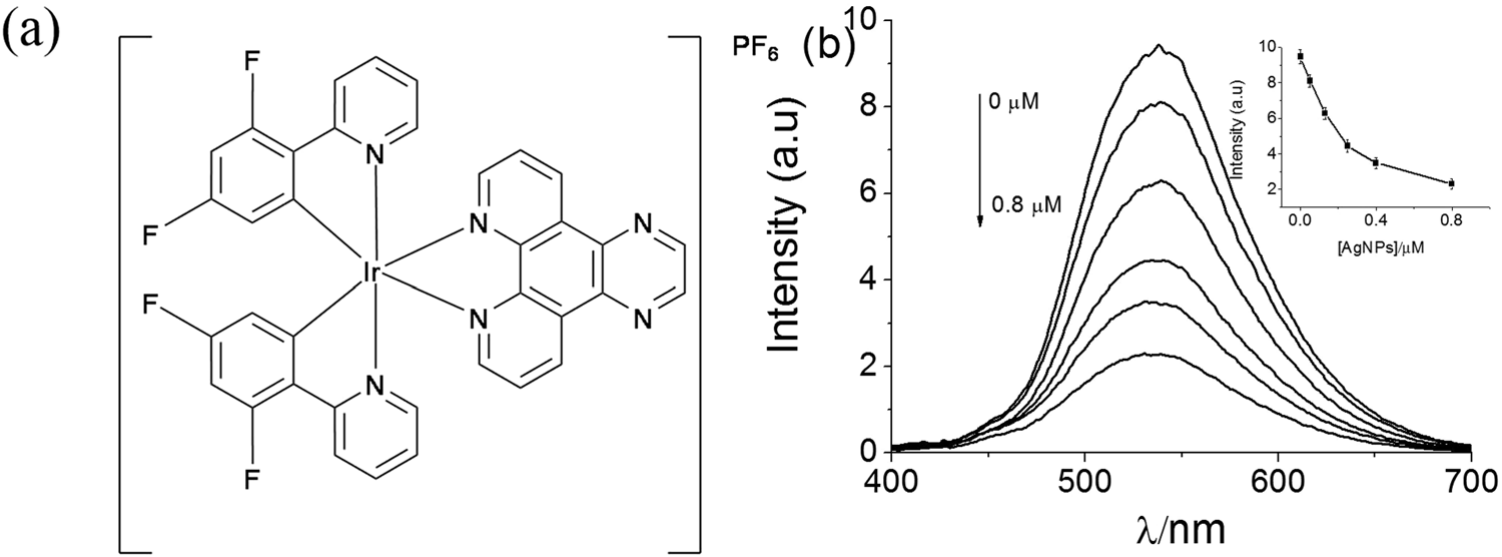
(**a**) Chemical structure of Ir(III) complex **1**. (**b**) Luminescence emission spectra 1.0 μM complex **1** in Tris-HNO_3_ buffer solution (pH 7.0) containing different concentrations of Ag nanoparticles. The inset is the luminescence intensity plotted against the Ag nanoparticles concentration (from top to bottom: 0, 0.05, 0.13, 0.25, 0.4, and 0.8 μM).

Silver nanoparticles (AgNPs) are a promising nanomaterial because of their remarkable properties, such as high extinction coefficient and surface plasmon resonance absorption^27^. AgNPs can also be oxidized by traces of Hg(II), forming Ag(I) and Ag/Hg amalgam^28, 29^. In addition, AgNPs are excellent quenchers of various luminescence probes, such as organic dyes and quantum dots (QDs)^30–32^. Meanwhile, Ir(III) complexes have arisen as promising tools to construct luminescent probes because of their large Stokes shifts, long-lived phosphorescence property, synthetic simplicity, as well as their readily tuned excitation and emission wavelengths^33, 34^. A number of strongly luminescent Ir(III) complexes have recently been synthesized by our research group as probes for various substances including small molecules, metal ions, proteins and enzymes^35–40^. However, to the best of our knowledge, the detection method for Hg(II) based on the combination of Ir(III) complex with AgNPs has not yet been reported so far. Herein, we have demonstrated a novel system based on the combination of luminescent complex **1** [Ir(F_2_-ppy)_2_(ppl)]^+^ (where F_2_-ppy = 2-(2,4-difluorophenyl)pyridine and ppl = pyrazino[2,3-*f*][1,10]phenanthroline) (Fig. 1a) and AgNPs for detecting Hg(II) ions through a turn-on luminescence mechanism.

**Figure 2:**
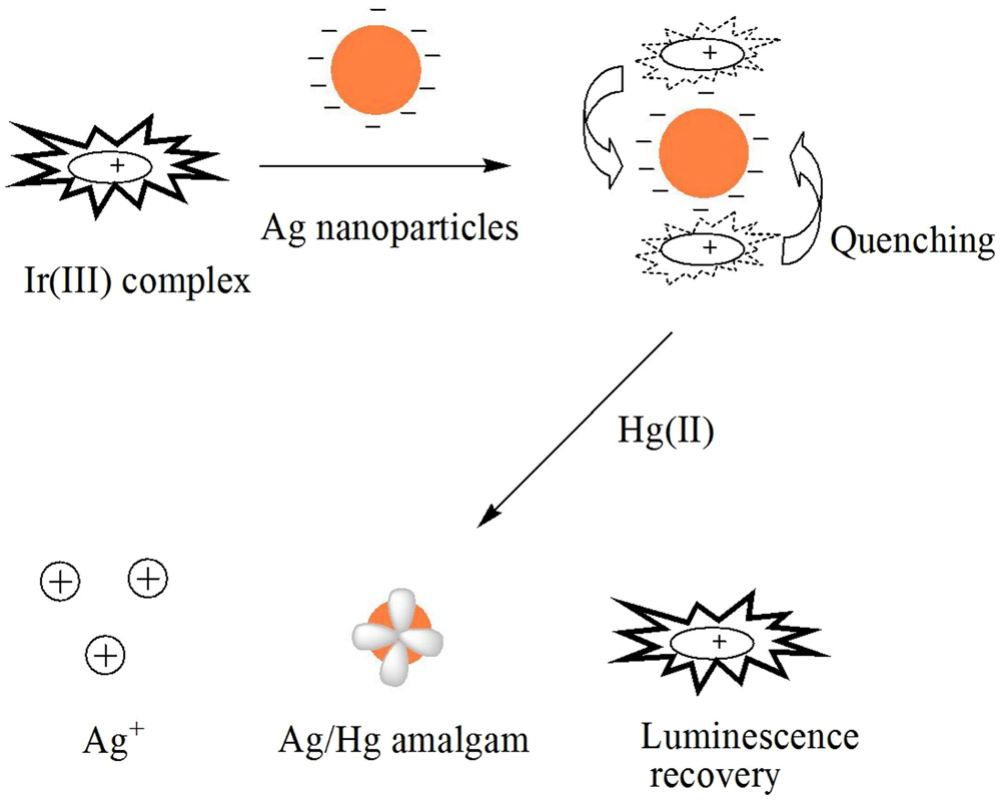
The illustration of the design rationale for the detection of Hg(II) using a luminescent sensor based on Ir(III) complex and Ag nanoparticles.

The detection mechanism of the assay is shown in Fig. 2. Initially, the positively charged complex **1** adsorbs to the surface of citrate-stabilized AgNPs through electrostatic interactions, leading to the formation of **1**/AgNP assemblies. The strong quenching abilities of AgNPs results in the luminescent quenching of **1** through the non-radiative energy transfer from **1** to AgNPs. Upon the addition of Hg(II) ions, the luminescence of the **1**/AgNP system is effectively recovered because AgNPs can be oxidized by Hg(II), resulting in the formation of soluble Ag(I). Subsequently, the elemental Hg formed interacts with the surface of AgNPs, followed by yielding the amalgam particles that eject the citrate molecules from their surface, which reduces the negative charge on the surface of AgNPs. Consequently, complex **1** is liberated from the AgNPs and the luminescence of complex **1** is recovered. The degree to which the luminescence is recovered is proportional to the Hg(II) ion concentration, allowing the accurate determination of Hg(II) ion concentration.

## Results

### Photophysical Properties of 1 and Signal Response to AgNPs

**Figure 3:**
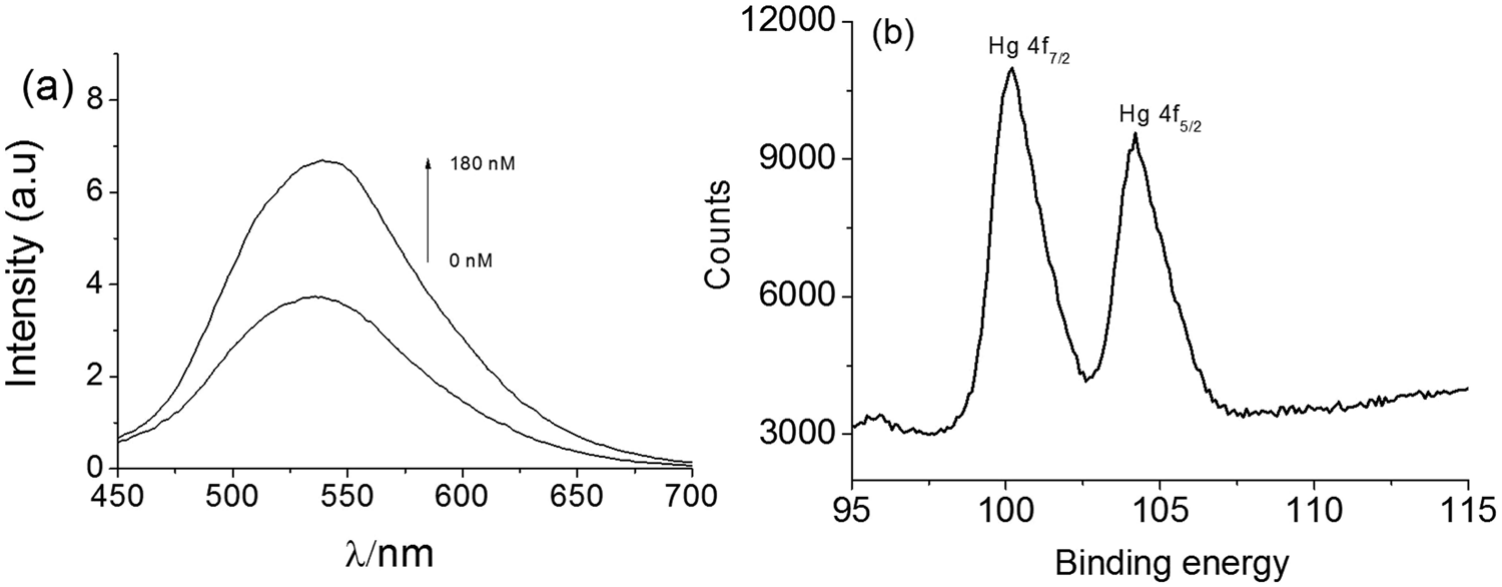
(**a**) Luminescence emission spectra of 1.0 μM complex **1** in Tris-HNO_3_ buffer solution (pH 7.0) containing 0.4 μM AgNPs and 0 or 180 nM of Hg(II). (**b**) XPS Hg 4 f regions spectra of AgNPs after interaction with Hg(II).

The detailed preparation and characterization of complex **1** is provided in the Supporting Information (Figures S1–S3, Table S1 (ESI†)). In aqueous solution, complex **1** emits a strong luminescence at 538 nm upon excitation at 300 nm. As shown in Fig. 1b, the addition of AgNPs led to a concentration-dependent quenching of the luminescence of complex **1**. We anticipated that the high quenching efficiency of AgNPs could improve the signal-to-noise ratio of the system when used to determine the concentration of a target analyte^41^. Then, the complex **1**/AgNP mixture was treated with Hg(II) (180 nM) at room temperature. As shown in Fig. 3a, the addition of Hg(II) caused the luminescence intensity of the **1**/AgNP system to increase. A control experiment found that the luminescence of complex **1** was not affected by Hg(II) in the absence of AgNPs (Figure S4 (ESI†)). Therefore, the restoration of the luminescence of the system was attributed to complex **1** being liberated from the AgNP surface upon the addition of Hg(II).

### Mechanism Validation

**Figure 4:**
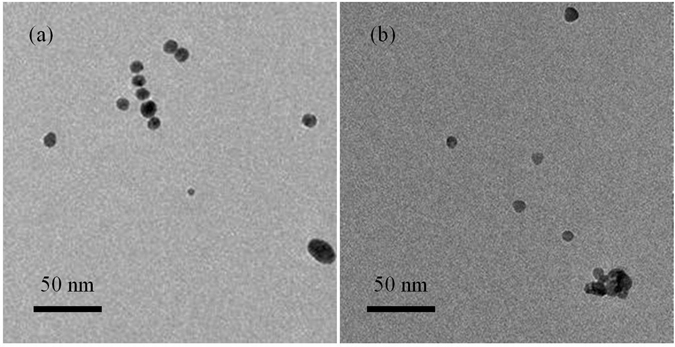
Transmission electron microscopy images of (**a**) AgNPs and (**b**) the AgNPs in the presence of Hg(II) ions.

The mechanism involved in the luminescence recovery process was investigated by acquiring UV–Vis absorbance spectra of a solution containing AgNPs in the absence and presence of Hg(II) ions. The characteristic and strong surface plasmon resonance peak was found at around 400 nm when only AgNPs were present^42, 43^. However, as can be seen in Figure S5 (ESI†), the absorption band of AgNPs gradually decreased accompanied by a slight blue shift from 397 to 387 nm upon increasing the concentration of Hg(II) ions. This phenomena was ascribed to the oxidation of AgNPs by Hg(II), leading to the formation of soluble Ag(I) and the subsequent generation of amalgam particles due to the deposition of elemental Hg on the AgNPs surface^37, 44–46^. The formation of elementary Hg was also confirmed by XPS analysis. As depicted in Figure S6 (ESI†), the survey spectrum revealed the presence of all possible elements, i.e. C, O, Ag and Hg. Peaks of Hg 4f_7/2_ are seen at 100.1 eV, indicating the formation of elemental Hg (Fig. 3b)^44^. Transmission electron microscopy (TEM) analysis further provided evidence that Hg(II) interacted with AgNPs. AgNPs in the absence of Hg(II) are mostly spherical with a narrow size distribution averaging around 9 nm in diameter (Fig. 4a). After treatment with Hg(II), the number of AgNPs reduced and some AgNPs aggregated together (Fig. 4b). The images are consistent with the conversion of AgNPs into soluble Ag(I) and the formation of Ag/Hg amalgam, which triggers NP aggregation.

The kinetics of the reaction between Hg(II) with the **1**/AgNP system were investigated by monitoring the change in luminescence as a function of time. As shown in Figure S7 (ESI†), the luminescence of this system increased quickly after the addition of 160 nM Hg(II)) and reached a constant level after three minutes. Therefore, a three-minute incubation period was used for the subsequent Hg(II) detection experiments.

### Signal response of 1/AgNP to Hg(II)

**Figure 5:**
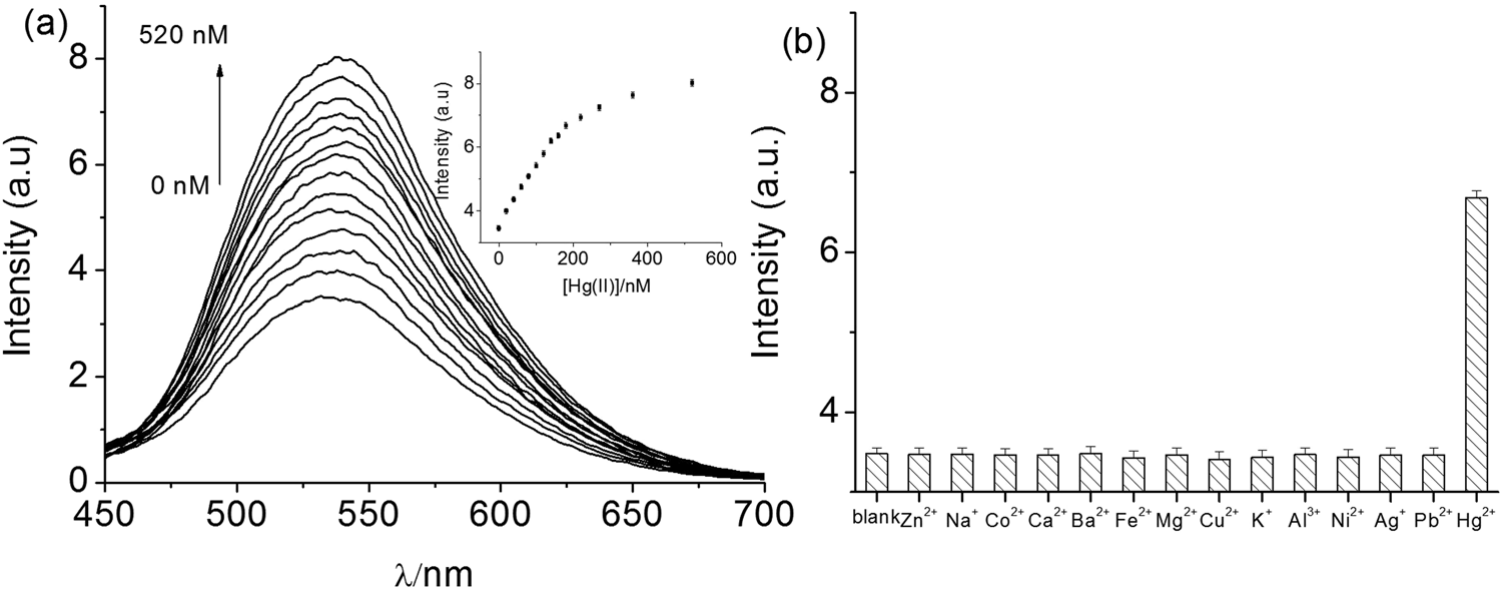
(**a**) Luminescence emission spectra of 1.0 μM complex **1** in Tris-HNO_3_ buffer solution (pH 7.0) containing 0.4 μM Ag nanoparticles and different concentrations of Hg(II) (from bottom to top: 0, 20, 40, 60, 80, 100, 120, 140, 160, 180, 220, 270, 360, and 520 nM). (**b**) Luminescence intensities of the **1**/AgNP system (1.0 μM **1** and 0.4 μM AgNPs in Tris-HNO_3_ at pH 7.0) in the presence of Hg(II) (180 nM) or interfering species (1 μM).

To demonstrate the application of the assay for the detection of Hg(II), various concentrations of Hg(II) were introduced to the **1**/AgNP system. As shown in Fig. 5a, the luminescence intensity of the **1**/AgNP sensor increased with an increasing concentration of Hg(II), which suggest that the addition of more Hg(II) can effectively increase the oxidization of AgNPs into Ag(I), with a concomitant increased formation of Ag/Hg amalgam. The luminescence enhancement displayed a linear range of Hg(II) concentration from 0 to 180 nM with a correlation coefficient of 0.997 (Figure S8 (ESI†)), indicating the potential capability of this system in quantitative analysis of Hg(II). Notably, a limit of detection (LOD) for Hg(II) was calculated to be 5 nM according to the equation *C*_lim_ = 3δ/*k*, which is lower than the maximum permissible level (10 nM) of Hg(II) in drinking water specified by the U.S. Environmental Protection Agency (EPA)^7, 46^. Additionally, the sensitivity of this proposed method is also found to be comparable to other reported methods for Hg(II) detection as summarized in Table S2 (ESI†)^6–8, 12, 13, 17,19–22,﻿﻿﻿ 47–53^.

### Selectivity of **1**/AgNP for Hg(II)

Selectivity is another important requirement for a probe. The luminescence intensity of **1**/AgNP was measured in the presence of 180 nM Hg(II) or 1 µM of potential interfering species including Zn^2+^, Na^+^, Co^2+^, Ca^2+^, Ba^2+^, Fe^2+^, Mg^2+^, Cu^2+^, K^+^, Al^3+^, Ni^2+^, Ag^+^ and Pb^2+^. The results showed that the luminescence of **1** was efficiently recovered when Hg(II) ions were added. Conversely, the addition of other testing species only elicits very little restoration of luminescence signal (Fig. 5b). The selectivity of the **1**/AgNP probe is presumably due to the fact that AgNPs only reacts with Hg(II) but not any of the other metal ions. Taken together, the results suggest that the **1**/AgNP sensor could detect Hg(II) with both high sensitivity and selectivity.

### Application of Hg(II) Detection Assay in Samples

To further investigate the feasibility of employing the proposed method to determine Hg(II) concentrations in real samples, standard recovery experiments were performed in natural water samples. Each water sample was passed through a 0.45 µm micro-pore membrane filter, followed by centrifuging for 10 min at 8000 rpm before being analyzed. As shown in Table 1, the recoveries of the Hg(II) ions were 97%–103%, suggesting that the sensor could potentially be used to determine Hg(II) ions in natural water samples.

**Table 1:** Hg(II) ion concentrations found in tap water samples using the standard addition method, and the recoveries and relative standard deviations (RSDs) of the Hg(II) added.

Sample	Detected (nM)	Added (nM)	Found (nM)	Recovery (%)	RSD (%)
Tap water	Not detected	50	48.5	97	2.3
	Not detected	100	103	103	2.2
	Not detected	150	153	102	2.5

## Conclusion

In conclusion, we have developed a novel **1**/AgNP-based system for rapid and label-free detection of Hg(II). The functionality of this protocol is highlighted by the quenching effect of AgNPs on the luminescence of complex **1**, followed by a subsequent turn-on luminescence upon the addition of Hg(II). The luminescence restoration complex **1** is attributed to the redox reaction of Hg(II) with AgNPs, which releases complex **1** from the AgNP surface. This method possess promising advantages including simple design, economic operation, as well as its “mix-and-detect” protocol which does not require chemical modification on AgNPs or complex **1**. In addition, this sensing platform exhibits excellent selectivity for Hg(II) over other metal ions, with the detection limit lower or comparable with the previously reported luminescence-based methods. It is anticipated that further optimization of this detection system may eventually be applied in the development of low-cost sensors for other metal ions and potentially be employed in environmental applications.

## Methods

### General experiments

The morphology of the AgNPs was investigated using transmission electron microscopy (TEM, Tecnai G2 20 S-TWIN Transmission Electron Microscope). For TEM measurements, the sample solutions were deposited on an Agar holey carbon-coated copper grid (300 mesh) and dried in a vacuum at room temperature before observation. XPS measurements were acquired with a Leybold Heraeus SKL 12 X-ray photoelectron spectrometer modified with a VG CLAM 4 multichannel hemispherical analyser. Mg Ka X-rays (1253.6 eV) were used as the excitation source to determine the binding energies of Hg (4 f) of the Ag/Hg nanoparticles. The samples were prepared by repeatedly spotting the purified Ag/Hg suspension on a silicon slice and allowed to dry. Mass spectrometry was performed at the Mass Spectroscopy Unit at the Department of Chemistry, Hong Kong Baptist University, Hong Kong (China). Melting points were determined using a Gallenkamp melting apparatus and are uncorrected. Deuterated solvents for NMR purposes were obtained from Armar and used as received. ^1^H and ^13^C NMR were recorded on a Bruker Avance 400 spectrometer operating at 400 MHz (^1^H) and 100 MHz (^13^C). ^1^H and ^13^C chemical shifts were referenced internally to solvent shift (acetone-*d*6: ^1^H, 2.05, ^13^C, 29.8). Chemical shifts (are quoted in ppm, the downfield direction being defined as positive. Uncertainties in chemical shifts are typically ±0.01 ppm for ^1^H and ±0.05 for ^13^C. Coupling constants are typically ±0.1 Hz for ^1^H-^1^H and ±0.5 Hz for ^1^H-^13^C couplings. The following abbreviations are used for convenience in reporting the multiplicity of NMR resonances: s, singlet; d, doublet; t, triplet; q, quartet; m, multiplet; br, broad. All NMR data was acquired and processed using standard Bruker software (Topspin).

### Synthesis of Complex **1**

Complex **1** was prepared according to a (modified) literature method^35^. Its structure was fully characterized by ^1^H-NMR, ^13^C-NMR, high resolution mass spectrometry (HRMS) and elemental analysis, and its photo physical properties were also tested. The synthesis of complex **1** is described as follows. Specifically, a suspension of [Ir_2_(F_2_-ppy)_4_Cl_2_] (0.2 mmol) and pyrazino[2,3-*f*][1,10]phenanthroline (0.42 mmol) in a mixture of DCM:methanol (1:1.2, 36 mL) was refluxed overnight under a nitrogen atmosphere. The resulting solution was then allowed to cool to room temperature, and filtered to remove unreacted cyclometallated dimer. Then, an aqueous solution of ammonium hexafluorophosphate (excess) was added into the filtrate, and the filtrate was reduced in volume by rotary evaporation until precipitation of the crude product occurred. The precipitate was then filtered and washed with several portions of water (2 × 50 mL) followed by diethyl ether (2 × 50 mL). The product was recrystallized in the acetonitrile:diethyl ether vapor diffusion to yield the titled compound as a red solid.

Complex **1**. Yield: 74%, ^1^H NMR (400 MHz, acetone-*d*_*6*_) δ 9.82 (d, 2 H), 9.37 (s, 2 H), 8.72 (d, 2 H), 8.39 (d, 2 H), 8.27–8.26 (m, 2 H), 8.02 (t, 1 H), 7,89 (d, 2 H), 7.07 (t, 2 H), 6.81 (t, 2 H), 5.92 (d, 2 H); ^13^C NMR (100 MHz, acetone-*d*_*6*_) δ 154.67, 153.89, 151.14, 149.42, 147.91, 140.72, 140.67, 136.61, 131.80, 129.36, 124.97, 124.65, 124.45, 114.97, 114.76, 100.17, 99.90, 99.63; MALDI-TOFHRMS: Calcd. for C_36_H_20_F_4_IrN_6_ [M–PF_6_]^+^: 805.1 Found: 805.3820. Anal.: (C_36_H_20_F_10_IrN_6_P) C, H, N: calcd. 45.53, 2.12, 8.85; found 45.44, 1.98, 8.89.

### Detection of Hg(II)

Complex **1** (1.0 μM) was mixed with AgNPs (0.4 μM) in each of a series of test tubes at room temperature. The mixture in each tube was buffered to pH 7.0 by adding Tris-HNO_3_ buffer. A different amount of stock Hg(II) solution was added to the mixture in each tube. The mixture was then allowed to equilibrate at room temperature for 3 min. Emission spectra were recorded in the 450−700 nm range using an excitation wavelength of 300 nm.

## Electronic supplementary material

ESI
