# Supplementary material for: Luminescent turn-on detection of Hg(II) via the quenching of an iridium(III) complex by Hg(II)-mediated silver nanoparticles
Source: Sci Rep. 2017 Jun 15;7:3620. doi: 10.1038/s41598-017-03952-x (PMC5472617; doi:10.1038/s41598-017-03952-x)
Supplement: Supplementary file 1 — ESI [file 41598_2017_3952_MOESM1_ESM.doc]

Supporting Information

**Luminescent turn-on detection of Hg(II) via the quenching of an iridium(III) complex by Hg(II)-mediated silver nanoparticles**

Jinshui Liu^a^, Kasipandi Vellaisamy^a^, Guanjun Yang^b^, Chung-Hang Leung^b^,* and Dik-Lung Ma^a^,*

^a^ Department of Chemistry, Hong Kong Baptist University, Kowloon Tong, Hong Kong, China.

^b^ State Key Laboratory of Quality Research in Chinese Medicine, Institute of Chinese Medical Sciences, University of Macau, Macao, China.

* Corresponding author:

Dr. Dik-Lung Ma, E-mail: edmondma@hkbu.edu.hk, Tel: (+852) 3411-7075, Fax: (+852) 3411-7348.

Dr. Chung-Hang Leung, E-mail: duncanleung@umac.mo, Tel: (+853)-8822-4688.

Contents:

[**General experimental.**](#__RefHeading___Toc11517)

[**Materials.**](#__RefHeading___Toc16155) [2](#__RefHeading___Toc20873) & 3

[**Figure S1**. ^1^H NMR spectra of complex **1**. 4](#__RefHeading___Toc4432)

[**Figure S2.** ^13^C NMR spectra of complex **1**. 5](#__RefHeading___Toc23685)

[**Figure S3.** MALDI-TOF HRMS spectra of complex **1**. 6](#__RefHeading___Toc30907)

[**Figure S4.** Luminescence of the complex **1** in the absence (a) and presence (b) of 5 μM Hg(II) ions. 7](#__RefHeading___Toc1038)

[**Figure S5.** UV−vis absorption spectra of AgNPs after the addition of different concentrations of Hg(II) ions (0−0.4 μM). 8](#__RefHeading___Toc6319)

[**Figure S6.** XPS survey spectrum of AgNPs after interacting with Hg(II).](#__RefHeading___Toc27984) 9

[**Figure S7.** Luminescence recovery from the Ir(III) complex **1**/Ag nanoparticles by Hg(II) as a function of time. 1](#__RefHeading___Toc1682)0

[**Figure S8.** Linear calibration plots of concentration of Hg(II) vs luminescence intensity of **1**. 1](#__RefHeading___Toc18766)1

[**Table S1.** Photophysical properties of iridium(III) complex **1** in acetonitrile at 298K. 1](#__RefHeading___Toc6591)2

[**Table S2.** Comparison of optical probes for the detection of Hg(II).](#__RefHeading___Toc6591) ………………………………............ 13

**Chemicals and materials.** Iridium chloride hydrate (IrCl_3_·xH_2_O) was purchased from Precious Metals Online (Australia). Other reagents were purchased from Sigma Aldrich (St. Louis, MO) and used as received. All of the reagents were of analytical grade and were used as received without further purification. All solutions were prepared in Milli-Q water under ambient conditions.

**Photophysical measurement.** Emission spectra and lifetime measurements for complex **1** were performed on a PTI TimeMaster C720 Spectrometer (Nitrogen laser: pulse output 337 nm) fitted with a 380 nm filter. Error limits were estimated: λ (±1 nm); τ (±10%); φ (±10%). All solvents used for the lifetime measurements were degassed using three cycles of freeze-vacthaw. Luminescence quantum yields were determined using the method of Demas and Crosby,^s1^ [Ru(bpy)_3_][PF_6_]_2_ in degassed acetonitrile as a standard reference solution (Φ_r_ = 0.062) and calculated according to the reported equation:

Φ_s_ = Φ_r_(*B*_r_/*B*_s_)(*n*_s_/*n*_r_)^2^(*D*_s_/*D*_r_) (1)

where the subscripts s and r refer to sample and reference standard solution respectively, *n* is the refractive index of the solvents, *D* is the integrated intensity, and Φ is the luminescence quantum yield. The quantity *B* was calculated by *B* = 1 – 10^–^*^AL^*, where *A* is the absorbance at the excitation wavelength and *L* is the optical path length.

**Synthesis of the AgNPs.** AgNPs were prepared according to the reported literature methods with slight modifications.^s2, s3^ Briefly, 0.10 mL of 0.1 M AgNO_3_ and 0.10 mL of 0.1 M trisodium citrate were added into 50 mL of water and placed in an ice bath. Then cold NaBH_4_ solution was added to it dropwise at very slow rate until the solution became vivid yellow. The resulting yellow colloidal silver solution was removed from the ice bath and stirred for 30 min, and then was stored at 4 °C overnight before further use. The size and morphology of the as-prepared AgNPs were characterized by transmission electron microscopy (TEM), which revealed an average diameter of 8–10 nm.

**Luminescence response of complex 1 towards AgNPs.** Complex **1** (1.0 μM) was added to various concentrations of AgNPs in Tris-HNO_3_ buffer (5 mM Tris-HNO_3_, pH 7.0), then their emission intensity were measured.


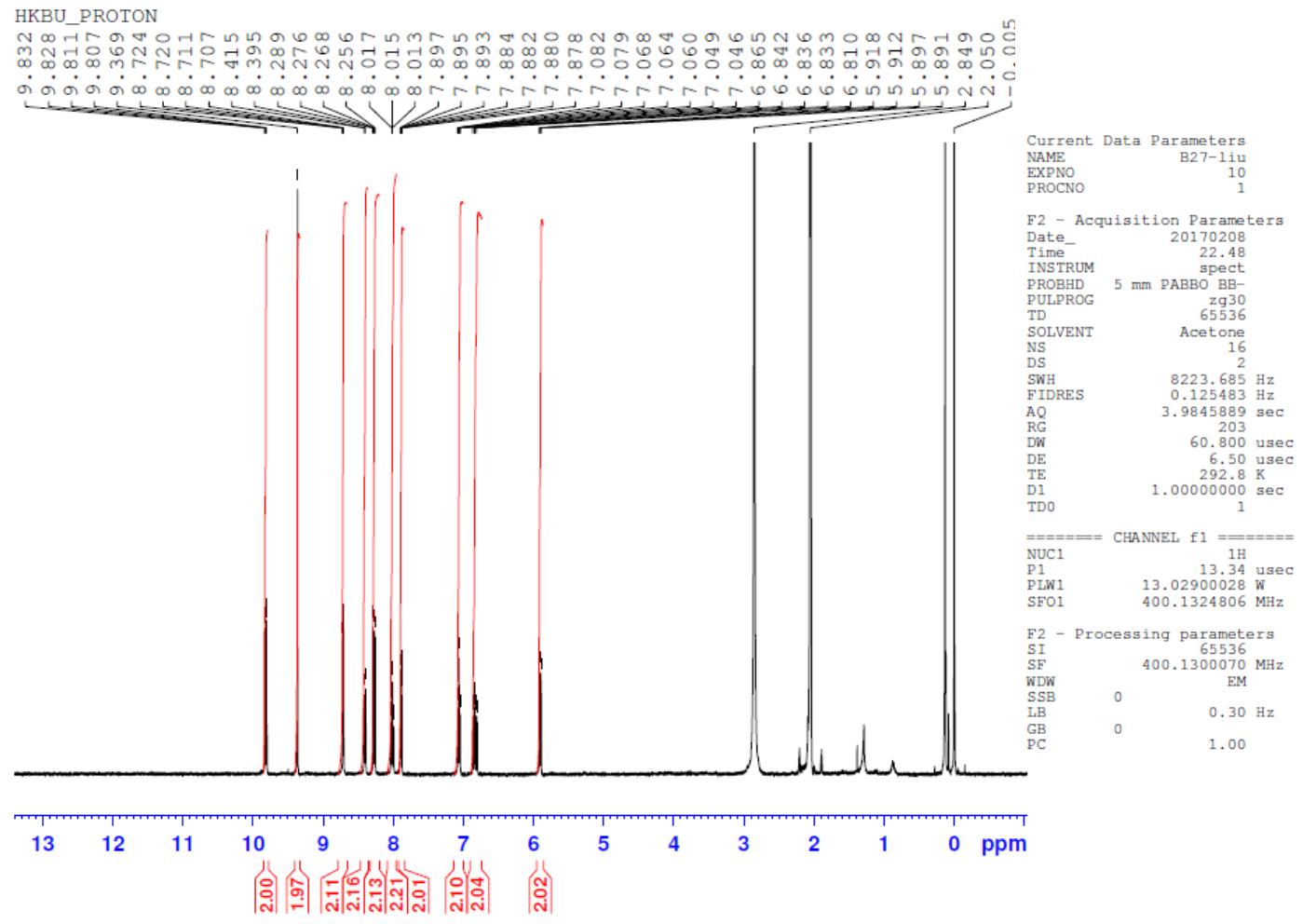


**Figure S1** ^1^H NMR spectra of complex **1**.


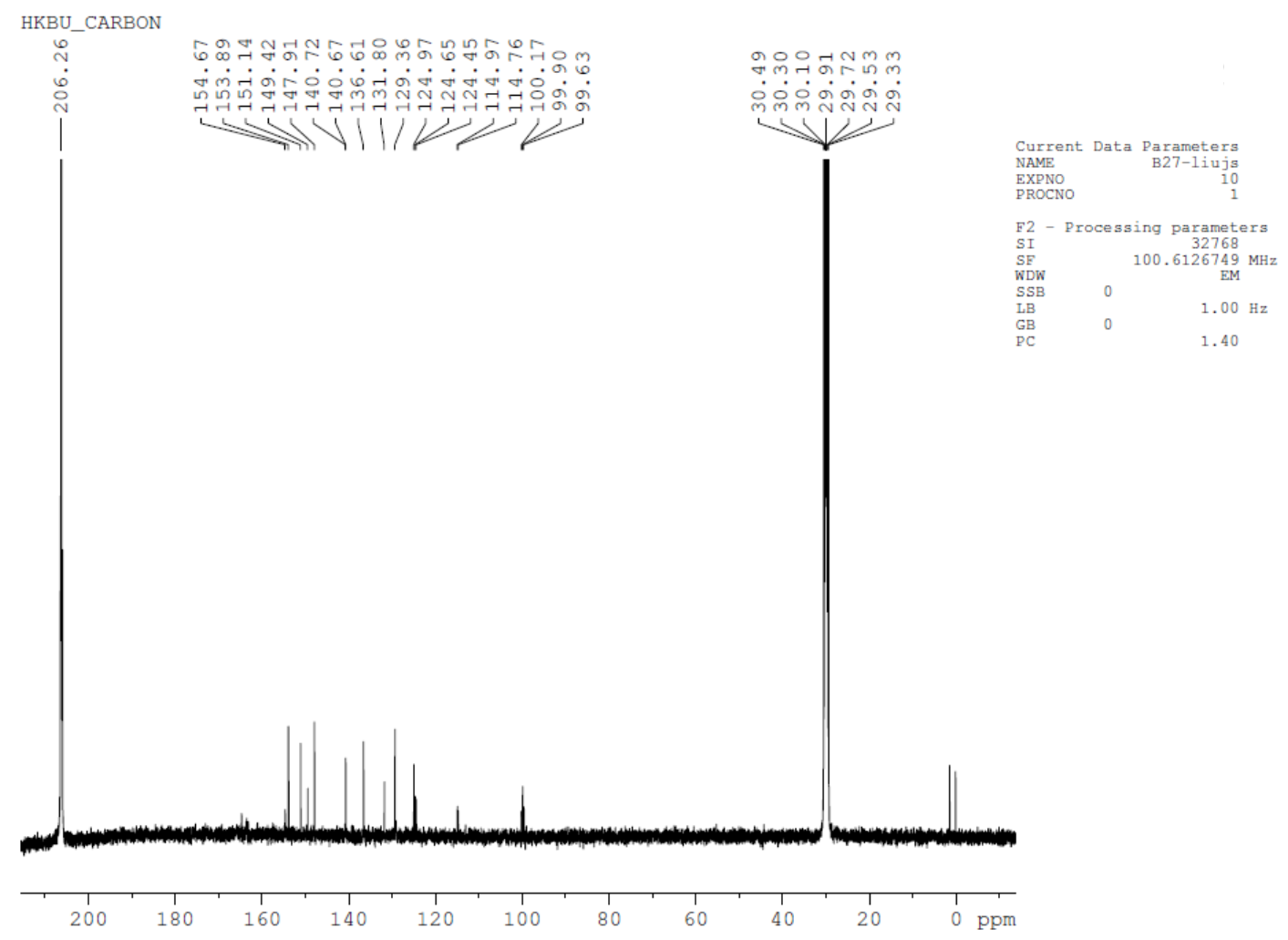


**Figure S2** ^13^C NMR spectra of complex **1**.

**Figure S3** MALDI-TOF HRMS spectra of complex **1**.

**Figure S4** Luminescence of the complex **1** in the absence (a) and presence (b) of 5 μM Hg(II) ions.

**Figure S5** UV−vis absorption spectra of AgNPs after the addition of different concentrations of Hg(II) ions (0−0.4 μM).

**Figure S6** XPS survey spectrum of AgNPs after interacting with Hg(II).

**Figure S7** Luminescence recovery from the Ir(III) complex **1**/Ag nanoparticles by Hg(II) as a function of time.

**Figure S8** Linear calibration plots of concentration of Hg(II) vs luminescence intensity of **1**.

**Table S1.** Photophysical properties of iridium(III) complex **1** in acetonitrile at 298K.

| Complex | Quantum yield | λ_em_ / nm | Lifetime / µs | UV/vis absorption  λ_abs_ / nm (ε / dm^3^mol^-1^cm^-1^) |
| --- | --- | --- | --- | --- |
| **1** | 0.31 | 538 | 4.135 | 256 (9.8 × 10^4^), 259 (1.1 × 10^4^) |

**Table S2** Comparison of optical probes for the detection of Hg(II).

| Hg(II) ion sensors | Methods | Detection limit | References |
| --- | --- | --- | --- |
| Gold Nanoparticles | Colorimetric | 2.9 nM | 7 |
| G-quadruplex conformational  transition | Colorimetric | 7.1 nM | 8 |
| DNA-Au NPs | Colorimetric | 500 nM | 44 |
| Quaternary ammonium-AuNPs | Colorimetric | 30 nM | 47 |
| Thiourea-AuNPs | Colorimetric | 193 nM | 48 |
| DNA-AuNPs | Colorimetric | 250 nM | 49 |
| Fluorogenic polymeric probe | Fluorometric | 6.6 μM | 6 |
| Ru-complex | Fluorometric | 2.34 nM, | 12 |
| Carbon quantum dots | Fluorometric | 100 nM | 13 |
| Rhodamine thiospirolactam | Fluorometric | 3 nM | 17 |
| Azo derivative sensor | Fluorometric | 46.5 nM | 19 |
| Carbon quantum dots | Fluorometric | 230 nM | 20 |
| Gold nanoclusters | Fluorometric | 58 nM | 21 |
| Silver nanocluster | Fluorometric | 4.5 nM | 22 |
| Carbon nanodots | Fluorometric | 4.2 nM | 43 |
| CdSe/ZnS quantum dots | Fluorometric | 10 nM | 45 |
| Silver nanoclusters | Fluorometric | 10 nM | 46 |
| Iridium(III) complex-AgNPs | Fluorometric | 5 nM | This work |

**References**

**10 nm**

s1. G. A. Crosby and J. N. Demas, *J. Phys. Chem.*, 1971, **75**, 991−1024.

s2. Y. Bhattacharjee and A. Chakraborty, *ACS Sustainable Chem. Eng.,* 2014, **2**, 149−2154.

s3. G. Wang, X. Zhua, H. Jiao, Y. Dong, and Z. Li, *Biosens. Bioelectron.*, 2012, **31**, 337–342.
